# Design Technology Co-Optimization Strategy for Ge Fraction in SiGe Channel of SGOI FinFET

**DOI:** 10.3390/nano13111709

**Published:** 2023-05-23

**Authors:** Shixin Li, Zhenhua Wu

**Affiliations:** 1 Institute of Microelectronics of the Chinese Academy of Sciences, Beijing 100029, China; lishixin@ime.ac.cn; 2 University of Chinese Academy of Sciences, Beijing 100049, China

**Keywords:** CMOS, FinFET, Silicon-On-Insulator (SOI), technology computer-aided design (TCAD), Design Technology Co-optimization (DTCO)

## Abstract

FinFET devices and Silicon-On-Insulator (SOI) devices are two mainstream technical routes after the planar MOSFET reached the limit for scaling. The SOI FinFET devices combine the benefits of FinFET and SOI devices, which can be further boosted by SiGe channels. In this work, we develop an optimizing strategy of the Ge fraction in SiGe Channels of SGOI FinFET devices. The simulation results of ring oscillator (RO) circuits and SRAM cells reveal that altering the Ge fraction can improve the performance and power of different circuits for different applications.

## 1. Introduction

The Moore’s Law predicts the scaling of semiconductor devices in very large scale integration circuits (VLSI). The process for planar metal oxide semiconductor field effect transistor (MOSFET) came to an end after reaching the limit for Dennard’s scaling [1]. In searching for the successor after the 20 nm technology node, FinFET devices and Silicon-On-Insulator (SOI) devices became two promising competitors [2,3]. FinFET devices provide enhanced gate controlling capability by the gate surrounding the channel [4], and SOI devices reduce the substrate leakage through buried oxide layers, providing lower delay and dynamic power consumption with no latch-up effect [5]. Eventually, FinFET won this competition due to its lower cost in fabrication. However, according to the International Roadmap for Devices and Systems (IRDS) 2022 [6], FinFET also faces the same problem of the scaling limit after the 7 nm node. As a potential candidate, FinFET on SOI devices were studied [7,8], for they share the advantages of both FinFET devices and SOI devices.

In many previous works, SiGe channel material is mainly used in PMOS rather than NMOS to enhance the hole mobility in PMOS devices [9,10,11]. The layer effects in SiGe channel are also studied [12,13]. The drawback of the SiGe channel in NMOS devices is the electron mobility degradation due to its high interface trap states [14,15], which can be eliminated in technology computer-aided design (TCAD) simulations [16]. Both FinFET devices with SiGe channels [17] and Silicon-Germanium-on-Insulator (SGOI) devices [10,18,19] are studied to improve the performance of the devices. However, there is a lack of studies on SGOI FinFET devices. Due to the existence of buried oxide layer, it is possible to fabricate FinFET devices on SOI substrates. For the same reason, the electrical isolations between source and drain region, and between the adjacent devices, can effectively suppress leakage and avoid latch-up effects.

In this work, we carried out a parasitic-aware DTCO flow of SGOI FinFET devices. From a common layout of 7 nm node FinFET, the devices are generated. We therefore employ state-of-the-art physics-based TCAD simulations and accurate parasitic extraction schemes to investigate the Ge content optimization strategy in the SiGe channels. Nine-stage ring oscillator (RO) circuits are built from the BSIM model of the devices and the parasitic parameters extracted from the devices. Subsequently, we run the SPICE transient simulation of the RO circuits, of which the results indicate the performance of each single RO circuit. Similarly, 6T-SRAM cells are also constructed in the same way. The read state, the hold state, and the write state of the SRAM cells are simulated by SPICE. These key performance indicators (KPIs) of both RO circuits and SRAM cells indicate the preferred optimizing strategy of the Ge content in the SiGe channels of SGOI FinFETs.

This article is arranged as follows. In Section 2, the device structure and simulation flow are introduced. In Section 3, the simulation results of RO circuits and SRAM cells are presented and discussed. Finally, the conclusions are given in Section 4.

## 2. Device Structure and Simulation Flow

**Figure 1:**
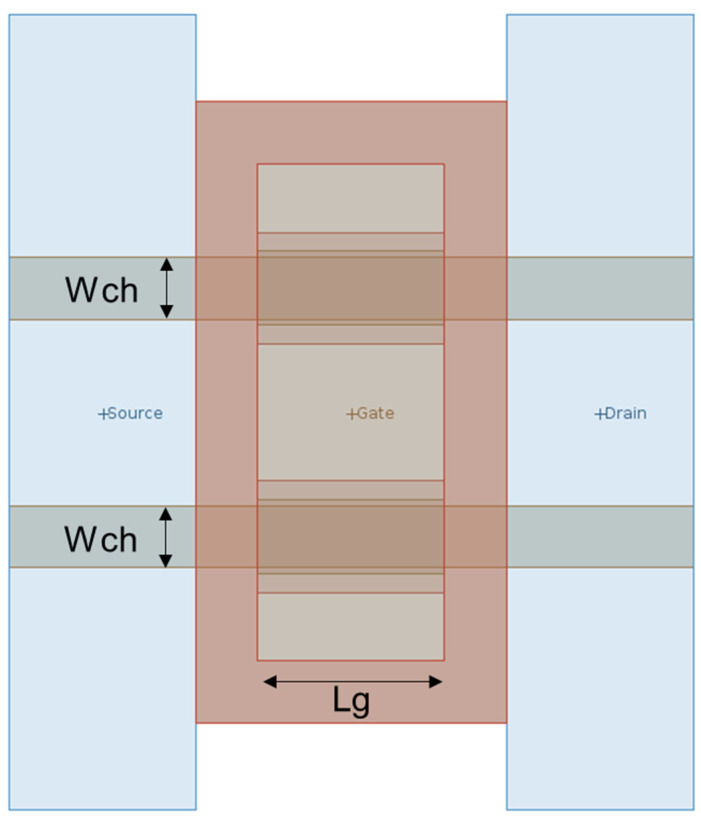
Layout of the layout-generated SGOI FinFET devices.

Figure 1 displays the layout of the SGOI FinFET devices in this study. As are labeled in the figure, there are 2 fins of 5 nm fin width (W_ch_) in each transistor, and the gate length (L_g_) is 15 nm.

**Figure 2:**
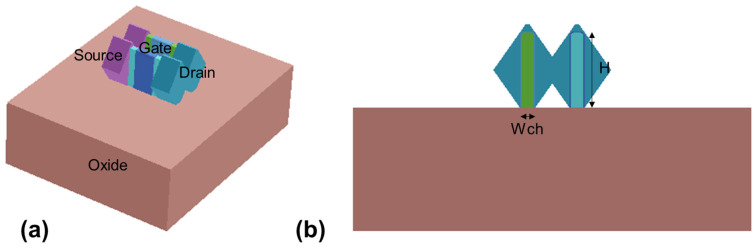
(**a**) The 3D device structure and (**b**) the cross-sectional view along the vertical axis of the simulated device.

Figure 2 shows the 3D structure and the cross-sectional view of the devices. The SiGe channel and the epi area are based on the oxide layer. As are listed in Table 1, the gate length (L_g_ = 15 nm), the gate oxide thickness (T_ox_ = 2 nm, 0.5 nm SiO_2_ +1.5 nm HfO_2_), the fin height (H = 30 nm), the spacer length (L_spacer_ = 5 nm), and the contact poly pitch (CPP = 40 nm) are consistent with the prediction of the international roadmap for devices and system (IRDS) 2018 [20] for 7 nm FinFET devices. The fins are with round corners of 1 nm radius to simulate realistic structures.

The channel experiences stress from two sources: the substrate and the S/D extent regions. As the substrate is oxide, the channel experiences little strain from the substrate. The S/D extent regions are SiGe, and are kept the same for all the devices.

It is possible for the fabrication of SGOI FinFET devices to be compatible with conventional CMOS technology, since it only requires the deposition of SiGe Fin on the SOI substrates.

**Figure 3:**
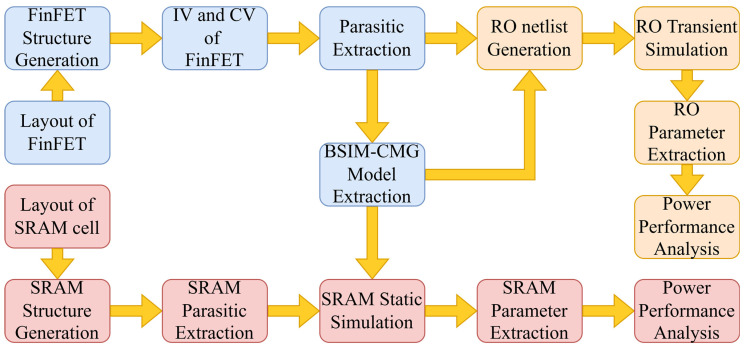
Simulation Flow of this Design Technology Co-optimization.

To evaluate the performance of the devices, we connected 9 inverters to each other to form a 9-stage ring oscillation circuit, and we constructed a standard 6T-SRAM. The simulation flow is illustrated in Figure 3. The simulation flow is illustrated in Figure 3. The simulation flow is separated in 3 parts as follows.

### 2.1. Device Structure Generation

The devices listed previously share a common layout as in Figure 1, and are generated as in Figure 2. For the devices only differ in doping type and Ge content in the SiGe channel, all the NMOS and PMOS devices share the same structure. The Ge content of the channels is altered by the parameter “ChannelMaterialComposition” when the SGOI devices are generated. The doping concentration in the channel is independent of the Ge content. The difference in Ge content affects the intrinsic parameters of the channel material such as density of states (DOS) and carrier mobility.

### 2.2. Device Simulation and Model Extraction

With help of GTS Minimos-NT, we simulate the transfer characteristics, output characteristics, and CV characteristics of the devices. The parasitic capacitances and resistances net of the devices are also extracted. The BSIM-CMG 110.0 model of the devices is extracted in an automatic process through Python script from the results of the characteristics simulation and the parasitic extraction.

### 2.3. Cell Circuits SPICE Simulation

For both single FinFET devices and cell circuits (RO and SRAM), the extracting process of the parasitic parameters is performed by GTS Minimos-NT. The netlist of the RO circuit for SPICE transient simulation is built from the extracted BSIM models and the parasitic parameters of the devices. By connecting the transistors and the parasitic capacitances and resistances, the netlist of a 9-stage RO circuit is built. By analyzing the transient curve of the output voltage variation with the input voltage, the power consumption and performance of these RO circuits are derived and evaluated.

The basic working flow for SRAM cells is almost the same, with the same model cards used. Differently, the SRAM cell structures are layout-generated, and the parasitic parameters of the SRAM cells are extracted as a whole system. The static and transient simulations of the SRAM cells are performed by SPICE. The power consumption and performance of the SRAM cells are analyzed and compared from the simulation results.

In order to improve the in-process and post-process accuracy of the simulation in this study, the following physical models are activated and considered in the TCAD simulation process:

1. The carrier transport and electrostatic potential problem is solved from the coupled Poisson’s equation and continuity equations, with the drift-diffusion model on.
2. The density gradient model is activated to improve the accuracy of the drift diffusion model in simulating nanoscale devices [21].
3. In order to accelerate the simulation process, the quasi-Fermi potential model is activated. As no continuity equation is to be solved, the size of the carrier equation system is reduced and the simulation time is shortened.
4. The bandgap narrowing model from Slotboom is included to modify the bandgap of silicon at a high doping level [22].
5. A ballistic transport model is considered because the gate length has reached the ballistic limit [23].
6. The carrier mobility for SiGe alloy is assumed to be linear and positively correlated to the Ge fraction in SiGe, which can be expressed as μ_SiGe_ = μ_Si_·(1 + C_0_x), where x denotes the material composition, and C_0_ is a constant factor that differs for electrons and holes.
7. The most important effect of altering the Ge content in this work is to modify the carrier mobility in the SiGe channel. The influence of mechanical stress and trap states is not taken into consideration of this simulation work.

## 3. Results and Discussion

### 3.1. Device Simulation Results

**Figure 4:**
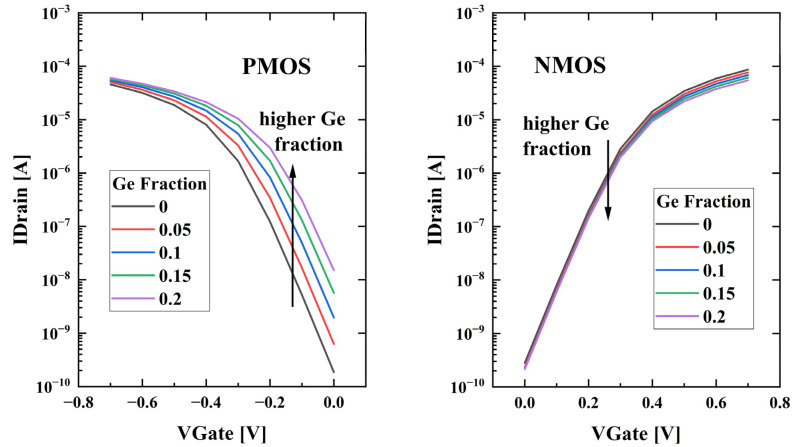
Saturated transfer characteristics of the devices from TCAD simulation.

The results of the saturated transfer characteristic simulation are illustrated in Figure 4. The linear transfer characteristics are simulated at V_Drain_ = ±0.05 V, and the saturated transfer characteristics are simulated at V_Drain_ = ±0.7 V. The subthreshold swing (SS) of the devices is extracted through constant current method at I_Drain_ = ±10^−7^ A. For NMOS devices, the transfer characteristics of different devices are essentially the same, and the SS at saturation is about 79 mV/decade. For PMOS devices, the SS at saturation is about 81 mV/decade. There is hardly any difference in SS and drain-induced barrier lowering (DIBL), which indicates the surrounding gate offers good control capability over the channel, and the buried oxide layer suppresses the leakage current.

**Figure 5:**
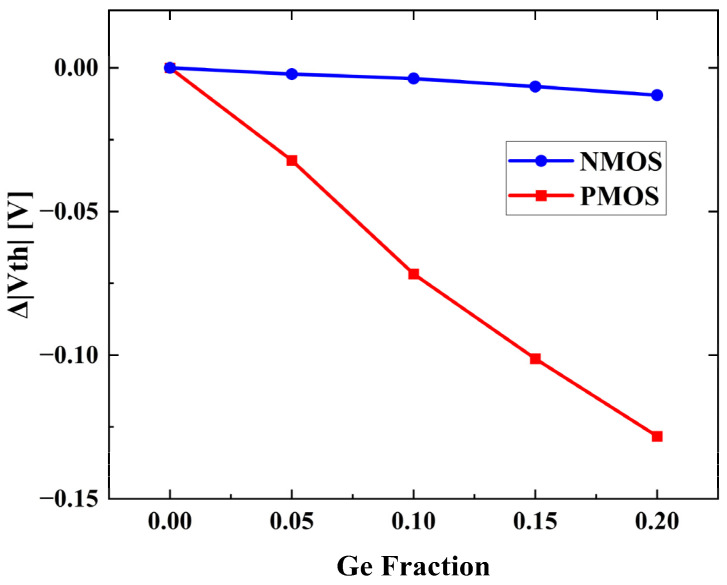
The dependence of threshold voltage on Ge fraction, with Ge fraction = 0 as reference.

Furthermore, the dependence of threshold voltage (V_th_) on Ge fraction of the simulated devices varies according to the doping type, as is displayed in Figure 5. The threshold voltages are extracted by finding the maximum curvature point on the curve. The relationship between the V_th_ and Ge fraction is approximately linear, and the slope of NMOS and PMOS appears to be different. The slope of NMOS devices hardly varies with Ge fraction, and is roughly −0.48 mV/Ge%. The slope of the absolute value of the threshold voltage variation with Ge fraction for PMOS devices is roughly −6.41 mV/Ge%. The CV characteristics of the devices are simulated at V_Drain_ = ±0.05 V, and V_Gate_ from −1.5 V to 1.5 V.

**Figure 6:**
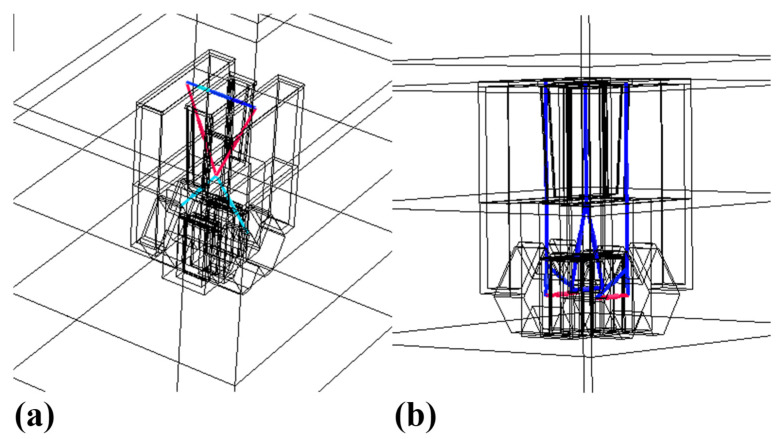
The extracted parasitic parameters in the SiGe NMOS. (**a**) parasitic capacitance net and (**b**) parasitic resistance net.

The parasitic capacitance and resistance net for each device is extracted. Figure 6a visualizes the extracted parasitic capacitance net in a single device, and Figure 6b presents the extracted parasitic resistance net. As is shown in Figure 6, the largest parasitic capacitance exists between gate and source (or drain). The largest resistance in a device is the source/drain epi resistance. The parasitic capacitance is a critical factor not only to the speed performance, but also to the power consumption of the circuit. By reducing the parasitic capacitance and resistance of a single device, the performance and power consumption of the circuit can be significantly optimized.

**Figure 7:**
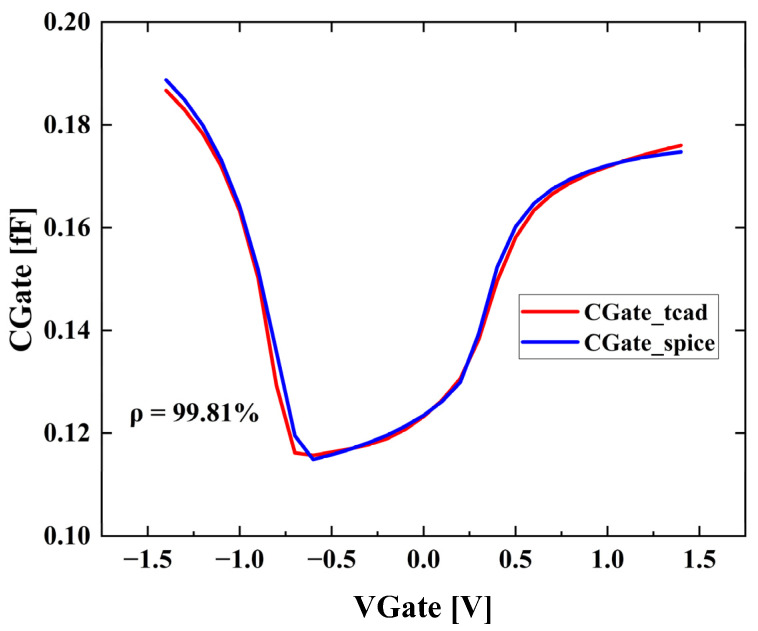
The CV characteristics of TCAD simulation and SPICE simulation.

Each BSIM model is extracted from 2 I_D_V_G_ curves, 3 I_D_V_D_ curves, 1 C_G_V_G_ curve, and the parasitic parameters. To estimate the accuracy of the extracted BSIM model, the IV and CV characteristics are re-simulated by SPICE simulator, and the correlation coefficients (ρ) are computed. Take CV characteristics for example, Figure 7 shows the TCAD and SPICE simulation results of the CV characteristics of the NMOS device with Silicon channel. The average correlation coefficient for CV characteristics of all devices is 0.9987, which indicates that the accuracy is guaranteed in the extraction process.

### 3.2. RO Results and Discussion

**Figure 8:**
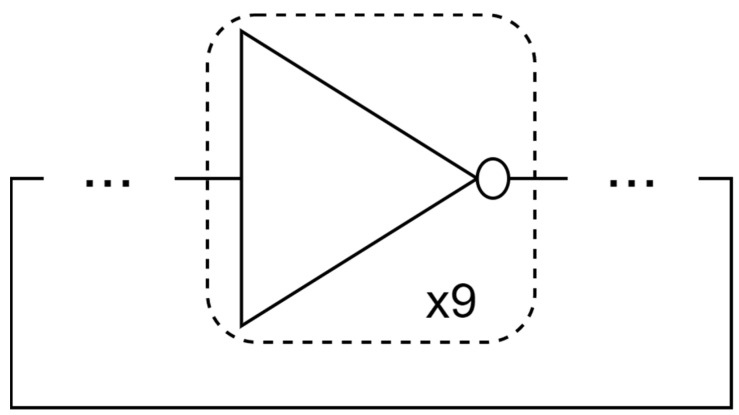
Equivalent circuit of the simulated 9-stage RO.

The 9-stage ring oscillator is constructed by connecting the gates and drains of 9 CMOS inverters to each other. The equivalent circuit of the simulated 9-stage RO is shown in Figure 8. To construct a ring oscillator circuit, the number of CMOS inverters needs to be an odd number. Selecting the 9-stage ring oscillator circuit is sufficient to evaluate the performance of the circuits, and is simple enough to accelerate the simulation process and shorten the simulation duration.

**Figure 9:**
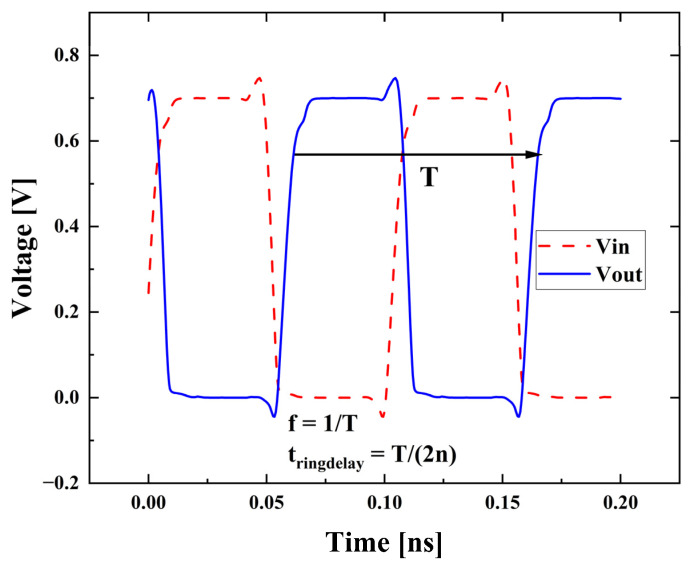
The transient simulation result of the RO consists of Si channel devices.

Figure 9 shows the transient simulation result of the RO circuit consists of Si channel devices with an initial perturbation applied. By extracting the neighboring highest and lowest points on the curve and multiplying by two, the period (T) of the RO circuit is calculated. The oscillation frequency (f) of the ring oscillation circuit is the reciprocal of the period, the transmission delay (t_ringdelay_) is the period divided by 2n (t_ringdelay_ = T/(2n)), and n is the number of stages of the RO circuit.

By analyzing the key performance indexes, the RO circuits are compared and evaluated to each other. Except for T, f, and t_ringdelay_, the average power consumption (P_avg_), effective capacity (C_eff_), effective resistance (R_eff_), and average operating current (I_DDA_) are also extracted from the transient curve. By averaging the operating current (I_DD_) over half a cycle (integrating the operating current over half a cycle, then divide the integration by the time), we derived the average operating current (I_DDA_). Likewise, the average power consumption (P_avg_) is obtained by averaging the product of operating current (I_DD_) and operating voltage (V_DD_) during half a cycle. The effective capacity (C_eff_) is calculated by the formula of C_eff_ = 2 t_ringdelay_ ∗ I_DDA_/V_DD_.

**Figure 10:**
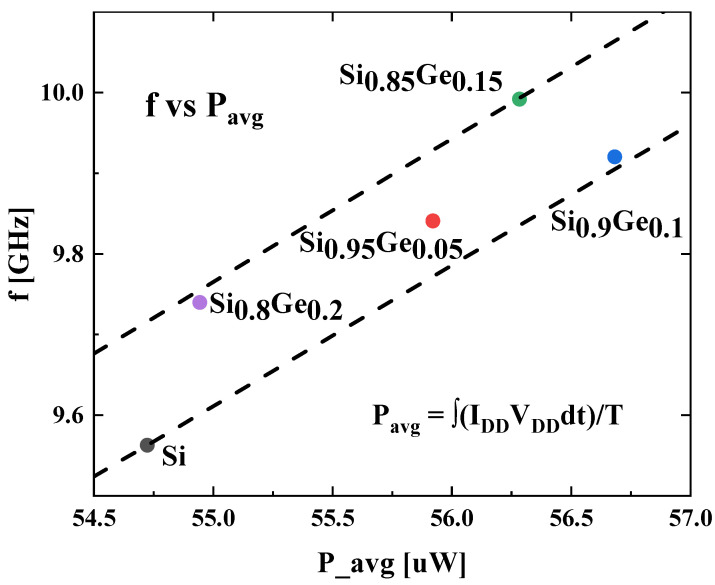
Oscillation frequency versus average power consumption.

The relationship between the RO oscillation frequency and the RO frequency is analyzed and compared, and the results are presented in Figure 10. The line passing through the point “Si” also passes through the coordinate origin, and so does the line pass through the point “Si_0.85_Ge_0.15_”. The other points lie in the region between the 2 lines. In general, the power consumption is positively correlated with the frequency, the circuit with higher Oscillation frequency has higher power consumption. Among the simulated devices, the Si_0.85_Ge_0.15_ device offers fastest speed (highest frequency of 9.99 GHz) with the second highest power consumption (56.28 μW), and the Si_0.95_Ge_0.05_ device provides medium speed with medium power consumption.

### 3.3. SRAM Results and Discussion

**Figure 11:**
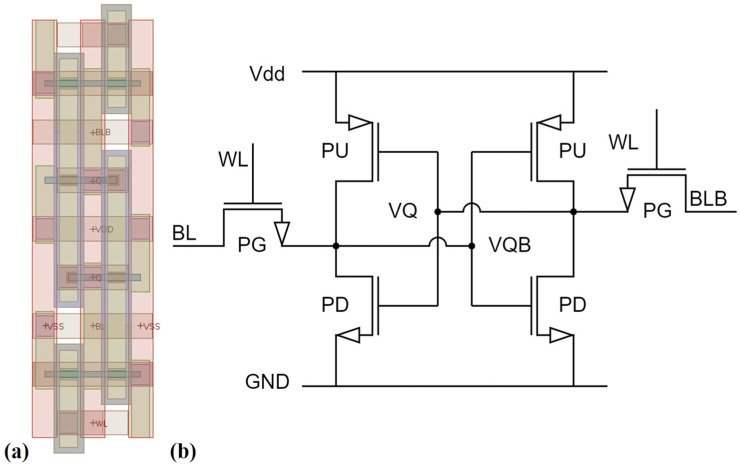
(**a**) Layout and (**b**) equivalent circuit of the simulated 6T-SRAM cell.

Subsequently, we constructed SRAM circuits in SPICE netlist from these devices. Figure 11a displays the layout of the basic 6T-SRAM cell. The equivalent SRAM cell is shown in Figure 11b, which is consist of 2 NMOS pass gate (PG) transistors, 2 NMOS pull down (PD) transistors, and 2 PMOS pull up (PU) transistors. Each SRAM cell is constructed of NMOS and PMOS devices with identical Ge fraction.

**Figure 12:**
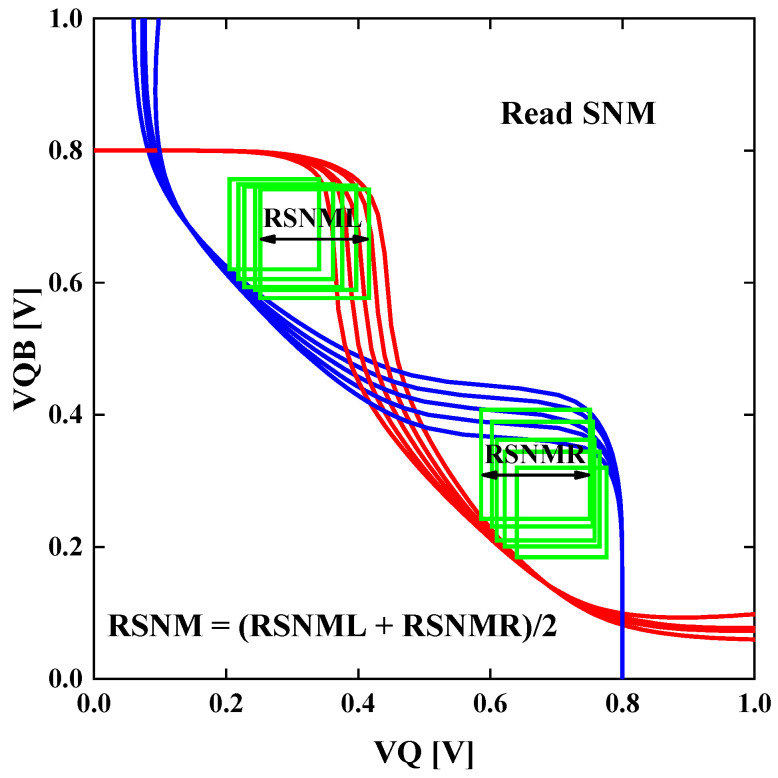
The simulated read voltage transfer curves and the method of extracting read SNM from the VTC.

The voltage transfer curves (VTC) of the circuits are simulated at V_DD_ = 0.8 V. The static read state, the static hold state, and the static write state of the SRAM cells are simulated, and the devices are evaluated by the KPIs of the SRAM cells. The basic method to extract static noise margin (SNM) from a voltage transfer curve of an SRAM, as is illustrated in Figure 12 for read SNM, is to find the largest square that can fit inside the area between the forward sweeping curve and the backward sweeping curve. The side length of which is the SNM we need. The read SNM and the hold SNM are both extracted through this method.

**Figure 13:**
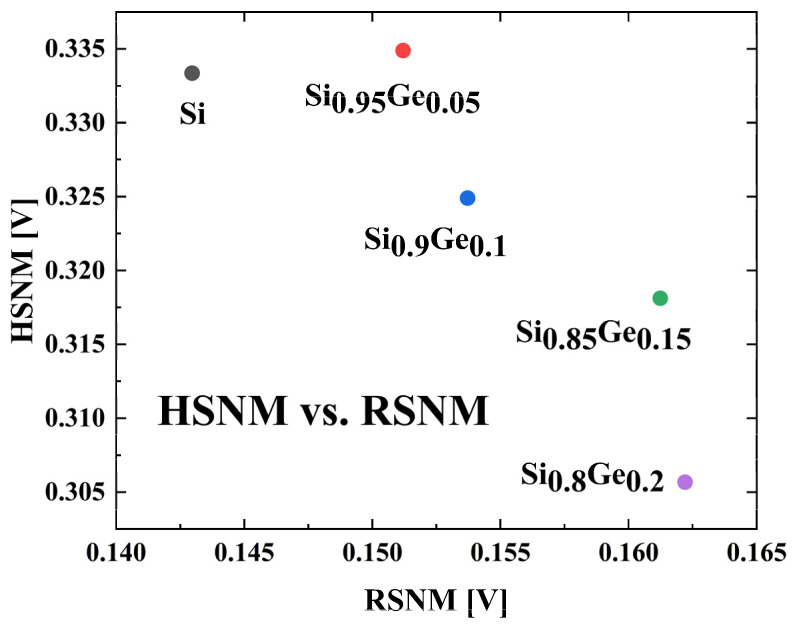
Hold SNM versus read SNM.

The read static noise margin and the hold static noise margin represents the stability of the SRAM during the read process and in hold state. Figure 13 shows the relationship between the read SNM and the hold SNM. The RSNM results are simulated by sweeping VQ forward while BL, BLB, and WL are at V_DD_ = 0.8 V. The HSNM results are simulated by sweeping VQ forward while BL, BLB at V_DD_ = 0.8 V, and WL at 0 V. For the goal of being able to resist greater noise, the device on the upper right part is more preferred.

For the read state, the read SNM is positively correlated with the Ge fraction in the SiGe channel, because higher drive current is presented in devices with higher Ge fraction. The SRAM with Si_0.8_Ge_0.2_ channel shows largest RSNM of 0.162 V. But for the hold state, the hold SNM reaches its maximum of 0.335 V at a Ge fraction of 0.05. Considering HSNM and RSNM together, the Si_0.95_Ge_0.05_ devices balance the best of the both indices.

**Figure 14:**
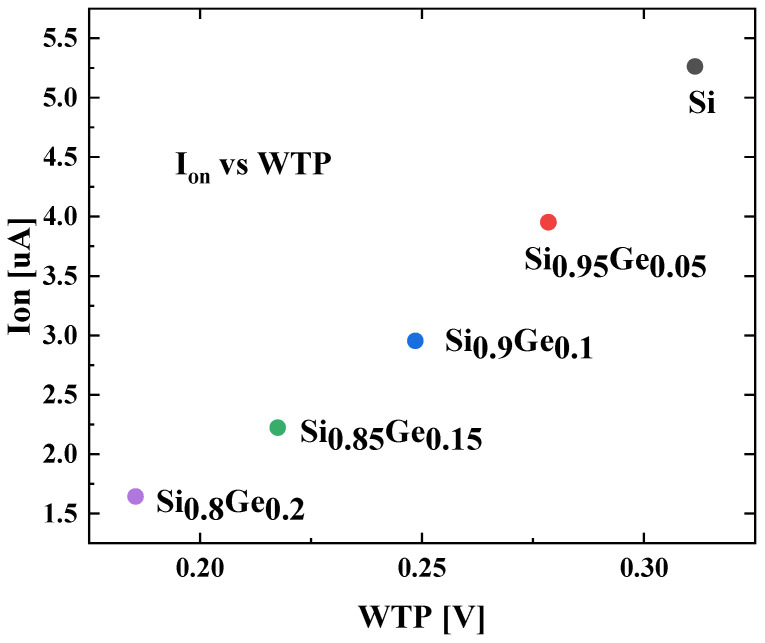
On-state current versus write trip point.

For write state simulation, the write trip point (WTP) and the on-state current are simulated. The WTP marks the voltage when the bit stored in SRAM flips, and the on-state current refers to the current of BL of SRAM when WL = 1. The relationship between the WTP and the on-state current is illustrated in Figure 14. The WTP and the on-state current are both negatively correlated with the Ge fraction in the SiGe channel. The Si devices offer the highest WTP of 0.31 V and the largest I_on_ of 5.26 μA. The Si_0.8_Ge_0.2_ devices provide the lowest WTP of 0.19 V and the smallest I_on_ of 1.64 μA.

## 4. Conclusions

In this paper, we performed a parasitic-parameter-involved Design Technology Co-optimization process on RO circuits and SRAM cells consist of SGOI FinFETs to optimize the Ge fraction in the SiGe channels, thereby to evaluate and select the SGOI FinFET devices architecture.

The simulation result shows the dependence of threshold voltage on channel Ge fraction differs in the doping type. The threshold voltage for NMOS devices decreases slightly with increase in Ge content, while the absolute value of the threshold voltage for PMOS devices decreases significantly. The Ge fraction dependent RO and the SRAM merits conclude that the optimal Ge fraction varies according to specific circuit structure.

## Figures and Tables

**Table 1:** Parameters and variables of devices in this work.

Parameter	Quantity	Value
L_g_	Gate Length	15 nm
W_ch_	Fin Width	5 nm
H	Fin Height	30 nm
T_ox_	Oxide Thickness	2 nm
L_spacer_	Spacer Length	5 nm
CPP	Contact Poly Pitch	40 nm
N_SD_	S/D Doping Concentration	3 × 20 cm^−3^
N_CH_	Channel Doping Concentration	3 × 15 cm^−3^
Channel Material	Channel Material	Si_1−x_Ge_x_
**Variable**	**Quantity**	**value**
x	Ge content	0, 0.05, 0.1, 0.15, 0.2

## Data Availability

The data presented in this study are available on request from the corresponding author.

## References

[1] Parvais B., Mercha A., Collaert N., Rooyackers R., Ferain I., Jurczak M., Subramanian V., Keersgieter A.D., Chiarella T., Kerner C. The device architecture dilemma for CMOS technologies: Opportunities & challenges of finFET over planar MOSFET. In Proceedings of 2009 International Symposium on VLSI Technology, Systems, and Applications.

[2] Chiarella T., Witters L., Mercha A., Kerner C., Rakowski M., Ortolland C., Ragnarsson L.-Å., Parvais B., De Keersgieter A., Kubicek S. (2010). Benchmarking SOI and bulk FinFET alternatives for PLANAR CMOS scaling succession. Solid-State Electron..

[3] Yan R.H., Ourmazd A., Lee K.F. (1992). Scaling the Si MOSFET: From bulk to SOI to bulk. IEEE Trans. Electron Devices.

[4] Hisamoto D., Lee W.-C., Kedzierski J., Takeuchi H., Asano K., Kuo C., Anderson E., King T.-J., Bokor J., Chenming H. (2000). FinFET-a self-aligned double-gate MOSFET scalable to 20 nm. IEEE Trans. Electron Devices.

[5] Yuan T., Buchanan D.A., Wei C., Frank D.J., Ismail K.E., Shih-Hsien L., Sai-Halasz G.A., Viswanathan R.G., Wann H.J.C., Wind S.J. (1997). CMOS scaling into the nanometer regime. Proc. IEEE.

[6] IEEE (2022). International Roadmap for Devices and Systems—More Moore White Paper, 2022 Edition.

[7] Chang J.B., Guillorn M., Solomon P.M., Lin C.H., Engelmann S.U., Pyzyna A., Ott J.A., Haensch W.E. Scaling of SOI FinFETs down to fin width of 4 nm for the 10nm technology node. In Proceedings of 2011 Symposium on VLSI Technology—Digest of Technical Papers.

[8] Xuejue H., Wen-Chin L., Kuo C., Hisamoto D., Leland C., Kedzierski J., Anderson E., Takeuchi H., Yang-Kyu C., Asano K. (2001). Sub-50 nm P-channel FinFET. IEEE Trans. Electron Devices.

[9] Verdonckt-Vandebroek S., Crabbe E.F., Meyerson B.S., Harame D.L., Restle P.J., Stork J.M.C., Johnson J.B. (1994). SiGe-channel heterojunction p-MOSFET's. IEEE Trans. Electron Devices.

[10] Cheng K., Khakifirooz A., Loubet N., Luning S., Nagumo T., Vinet M., Liu Q., Reznicek A., Adam T., Naczas S. High performance extremely thin SOI (ETSOI) hybrid CMOS with Si channel NFET and strained SiGe channel PFET. In Proceedings of 2012 International Electron Devices Meeting.

[11] Verdonckt-Vandebroek S., Crabbe E.F., Meyerson B.S., Harame D.L., Restle P.J., Stork J.M.C., Megdanis A.C., Stanis C.L., Bright A.A., Kroesen G.M.W. (1991). High-mobility modulation-doped SiGe-channel p-MOSFETs. IEEE Electron Device Lett..

[12] Li Y., Zhao F., Cheng X., Liu H., Zan Y., Li J., Zhang Q., Wu Z., Luo J., Wang W. (2021). Four-Period Vertically Stacked SiGe/Si Channel FinFET Fabrication and Its Electrical Characteristics. Nanomaterials.

[13] Li Y., Zan Y., Cheng X., Zhao F., Liu H., Wang W. (2022). Si0.5Ge0.5 channel introduction technique for the preparation of high mobility FinFET device. Mater. Sci. Semicond. Process..

[14] Hutin L., Cassé M., Royer C.L., Damlencourt J.F., Pouydebasque A., Xu C., Tabone C., Hartmann J.M., Carron V., Grampeix H. 20nm gate length trigate pFETs on strained SGOI for high performance CMOS. In Proceedings of 2010 Symposium on VLSI Technology.

[15] Oh J., Lee S.H., Min K.S., Huang J., Min B.G., Sassman B., Jeon K., Loh W.Y., Barnett J., Ok I. SiGe CMOS on (110) channel orientation with mobility boosters: Surface orientation, channel directions, and uniaxial strain. In Proceedings of 2010 Symposium on VLSI Technology.

[16] Yao J., Li J., Luo K., Yu J., Zhang Q., Hou Z., Gu J., Yang W., Wu Z., Yin H. (2018). Physical Insights on Quantum Confinement and Carrier Mobility in Si, Si0.45Ge0.55, Ge Gate-All-Around NSFET for 5 nm Technology Node. IEEE J. Electron Devices Soc..

[17] Guo D., Karve G., Tsutsui G., Lim K.Y., Robison R., Hook T., Vega R., Liu D., Bedell S., Mochizuki S. FINFET technology featuring high mobility SiGe channel for 10nm and beyond. In Proceedings of 2016 IEEE Symposium on VLSI Technology.

[18] Tezuka T., Sugiyama N., Mizuno T., Takagi S. (2003). Ultrathin body SiGe-on-insulator pMOSFETs with high-mobility SiGe surface channels. IEEE Trans. Electron Devices.

[19] Tezuka T., Nakaharai S., Moriyama Y., Sugiyama N., Takagi S. (2005). High-mobility strained SiGe-on-insulator pMOSFETs with Ge-rich surface channels fabricated by local condensation technique. IEEE Electron Device Lett..

[20] IEEE (2018). International Roadmap for Devices and Systems—More Moore White Paper, 2018 Edition.

[21] Ancona M.G., Tiersten H.F. (1987). Macroscopic physics of the silicon inversion layer. Phys. Rev. B Condens. Matter..

[22] Slotboom J.W., de Graaff H.C. (1976). Measurements of bandgap narrowing in Si bipolar transistors. Solid-State Electron..

[23] Erlebach A., Lee K., Bufler F.M. Empirical ballistic mobility model for drift-diffusion simulation. In Proceedings of 2016 46th European Solid-State Device Research Conference (ESSDERC).

